# Optimal drug regimens for primary biliary cirrhosis: a systematic review and network meta-analysis

**DOI:** 10.18632/oncotarget.4528

**Published:** 2015-06-19

**Authors:** Gui-Qi Zhu, Sha Huang, Gui-Qian Huang, Li-Ren Wang, Yi-Qian Lin, Yi-Ming Wu, Ke-Qing Shi, Jiang-Tao Wang, Zhi-Rui Zhou, Martin Braddock, Yong-Ping Chen, Meng-Tao Zhou, Ming-Hua Zheng

**Affiliations:** ^1^ Department of Infection and Liver Diseases, Liver Research Center, The First Affiliated Hospital of Wenzhou Medical University, Wenzhou, China; ^2^ School of the First Clinical Medical Sciences, Wenzhou Medical University, Wenzhou, China; ^3^ Renji School of Wenzhou Medical University, Wenzhou, China; ^4^ Institute of Hepatology, Wenzhou Medical University, Wenzhou, China; ^5^ Department of Radiation Oncology, Fudan University Shanghai Cancer Center; Department of Oncology, Shanghai Medical College, Fudan University, Shanghai, China; ^6^ Global Medicines Development, AstraZeneca R&D, Loughborough, United Kingdom; ^7^ Department of Hepatobiliary Surgery, The First Affiliated Hospital of Wenzhou Medical University, Wenzhou, China

**Keywords:** primary biliary cirrhosis, UDCA-based therapy, adverse events, network meta-analysis, indirect comparison

## Abstract

**Objective:**

Most comprehensive treatments for PBC include UDCA, combination of methotrexate (MTX), corticosteroids (COT), colchicine (COC) or bezafibrate (BEF), cyclosporin A (CYP), D-penicillamine (DPM), methotrexate (MTX), or azathioprine (AZP). Since the optimum treatment regimen remains inconclusive, we aimed to compare these therapies in terms of patient mortality or liver transplantation (MOLT) and adverse event (AE).

**Methods:**

We searched PubMed, Embase, Scopus and the Cochrane Library for randomized controlled trials until August 2014. We estimated HRs for MOLT and ORs for AE. The sensitivity analysis based on dose of UDCA was also performed.

**Results:**

The search identified 49 studies involving 12 different treatment regimens and 4182 patients. Although no statistical significance can be found in MOLT, COT plus UDCA was ranked highest for efficacy outcome amongst all the treatment regimes. While for AEs, compared with OBS or UDCA, monotherapy with COC (OR 5.6, *P* < 0.001; OR 5.89, *P* < 0.001), CYP (OR 3.24, *P* < 0.001; OR 3.42, *P* < 0.001), DPM (OR 8.00, *P* < 0.001; OR 8.45, *P* < 0.001) and MTX (OR 5.31, *P* < 0.001; OR 5.61, *P* < 0.001) were associated with statistically significant increased risk of AEs. No significant differences were found for other combination regimes. Effect estimates from indirect comparisons matched closely to estimates derived from pairwise comparisons. Consistently, in the sensitivity analysis, results closely resembled our primary analysis.

**Conclusions:**

COT plus UDCA was the most efficacious among treatment regimens both for MOLT and AEs.

## INTRODUCTION

Primary biliary cirrhosis (PBC) is a chronic progressive inflammatory autoimmune- mediated liver disease that primarily affects middle-aged women with a gender ratio of 1:10 [1]. The annual incidence of primary biliary cirrhosis ranges from 1 to 49 persons per million, and the prevalence was estimated between 7 to 402 persons per million [2].

So far, some single or combined therapies have been studied by randomized controlled trials (RCTs). However, which treatment regime is optimal for patients with PBC remains controversial. Although UDCA is the only medical treatment that has received U.S. Food and Drug Administration approval [3], the effects of UDCA evaluated by several clinical studies have yielded conflicting results. Some randomized clinical trials (RCT) have confirmed that UDCA is an effective medical treatment for the disease [4–7]. Also, UDCA has been shown to improve not only symptoms, liver enzymes, and liver histology, but also patient survival [8–9]. Conversely, two comprehensive traditional meta-analyses which include 16 recent RCTs concluded that the use of UDCA did not demonstrate any benefit on mortality and mortality or live transplantation (MOLT) [10–11]. Furthermore, PBC disease continued to progress in many patients who do not show complete response during UDCA therapy, hence, additional medical treatment or other drugs are urgently required.

Other drugs for the treatment of PBC have been studied for the past decade as single agents or adjuvant medications, which are mainly immunomodulatory and other agents, such as colchicine (COC), cyclosporin A (CYP), D-penicillamine (DPM), methotrexate (MTX), corticosteroids (COT), bezafibrate (BEF), or azathioprine (AZP). Although some of these drugs when administered as monotherapy showed little benefit for patients with PBC in clinical outcomes from several conventional meta-analyses, we are still unable to identify the clinical effects of drugs evaluated in traditional meta-analysis with a small number of included studies. Moreover, addition of these treatments to UDCA was evaluated by some studies. Combination therapy with UDCA and COC in patients with PBC has been evaluated in a small study and in a large double blind, placebo-controlled study [12–13]. Results from the Japanese study (12) indicate no benefits from the addition of COC to UDCA, whilst the French RCT (13) suggests that the addition of COC to UDCA provides only a marginal advantage over UDCA monotherapy. The role of combination therapy with MTX and UDCA were evaluated by Leung et al, which showed a clinical improvement compared with that predicted by the Mayo model [3, 14]. However, another RCT evaluating a combination of MTX plus UDCA demonstrated that there was no improvement in symptoms and the regime may have been associated with substantial toxicity. Consistently, the clinical outcomes of other additional therapies (COT or BEF) to UDCA still remain unclear or yield inconsistent results [2, 15].

Therefore, in order to attempt to resolve this issue, which theoretically may be answered by conducting a very large clinical trial with multiple comparator arms, of conflicting results in PBC treatments for patients, we performed the network meta-analysis, which allows us to integrate direct and indirect comparisons to simultaneously compare several treatments [16–18]. The objective of network meta-analysis was to apply the established methodology used in network meta-analysis to an area of clinical practice where no such previous studies have existed. In such cases, our aims were to summarize a much broader evidence base and to indirectly compare the relative efficacy and safety of these treatments with most comprehensive therapies (AZP, MTX, COT, COC, CYP, DPM, UDCA, MTX plus UDCA, COT plus UDCA, COC plus UDCA or BEF plus UDCA) for patients with PBC.

## RESULTS

### Study characteristics

**Figure 1:**
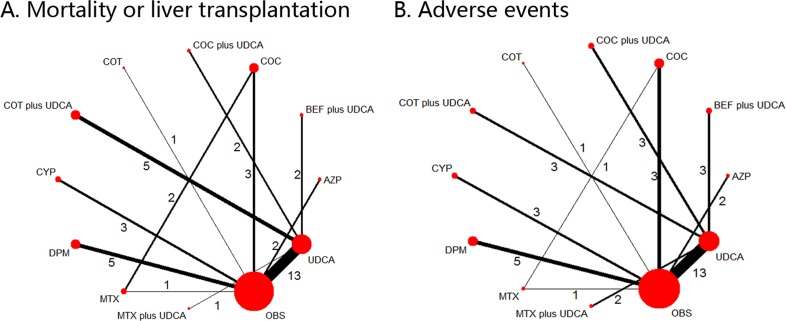
Network of the comparisons for the Bayesian network meta-analysis The numbers along the link lines indicate the number of trials or pairs of trial arms. Lines connect the interventions that have been studied in head-to-head (direct) comparisons in the eligible controlled trials. The width of the lines represents the cumulative number of trials for each comparison and the size of every node is proportional to the number of enrolled participants (sample size). COC: colchicine; BEF: bezafibrate; COT: corticosteroids; MTX: methotrexate; UDCA: ursodeoxycholic acid; CYP: cyclosporin A; DPM: D-penicillamine; AZP: azathioprine; OBS: observation. **A.** Mortality or liver transplantation; **B.** Adverse events.

3133 studies were identified for review of title and abstract (Supplementary 1). After the initial screening, we retrieved the full text of potentially eligible articles for detailed assessment, 3084 articles were excluded. We included forty-nine eligible studies for meta-analysis, with a total of 4182 patients who received one of the eleven treatment strategies including monotherapy with UDCA, AZP, MTX, COT, COC, CYP, DPM, combination of MTX plus UDCA, COT plus UDCA, COC plus UDCA, BEF plus UDCA or observation (Figure 1). The duration of treatment ranged from three months to ten years, and the mean age of trial participants was 55.3 years and ranged from 24 to 79 years. Most trials (47 [96%] of 31) were two-grouped studies and only two [19–20] were three-grouped studies and mean study sample was 41.8 patients per group (minimum-maximum 4-176). For the primary outcome, twelve unique comparisons were available for 40 [2, 4–5, 7, 15, 20–37] different trials. In terms of outcome of safety, there were also 40 [2–5, 7, 12–13, 20–32, 34–35, 38–55] trials providing data for twelve unique comparisons. Table 1 summarizes the characteristics of the included forty-nine studies. Supplementary 2 shows the quality assessment parameter assessed by the Cochrane Risk of Bias tool, which was rated as good, even though some studies did not reported details regarding randomization and allocation concealment.

**Table 1:** Characteristics of included studies

Author (Year)	Country	Mean age (range)	Treatment/Control	Dose	Treatment duration	Study size	Mortality or liver transplantation (%)	Adverse events (%)
							Treatment/Control	Treatment/Control
Itakura (2004)	Japan	57 (47–67)	BEF plus UDCA/UDCA	BEF: 400 mg per day; UDCA: 600 mg per day	6 months	16	8/6	11/14
Iwasaki (a) (2008)	Japan	56 (45–67)	BEF plus UDCA/UDCA	BEF: 400 mg per day; UDCA: 600 mg per day	52 weeks	22	0/0	42/10
Iwasaki (b) (2008)	Japan	56(28–75)	BEF/UDCA	BEF :400 mg daily orallyUDCA;600 mg daily	52 weeks	45	NR/NR	
Kanda (2003)	Japan	57 (52–72)	BEF plus UDCA/UDCA	BEF: 400 mg per day; UDCA: 600 mg per day	6 months	22	11/11	18/9
Leuschner (1999)	German	50 (40–60)	COT plus UDCA/UDCA	COT: 3 mg three times daily; UDCA: 10–15 mg/kg/day in three divided doses	2 years	40	5/5	10/5
Combes (2005)	United States	53 (42–60)	MTX plus UDCA/UDCA	MTX: in four doses over 48 hours (total dose 10 mg/week); UDCA: 500 mg per day	117 months	265	NR/NR	10/9
Gonzalez-Koch (1997)	Chile	53 (45–75)	MTX plus UDCA/UDCA	UDCA: 13–15mg/kg/day MTX: 0.25mg/kg/week	48 weeks	25	12/10	25/8
Lindor (1995)	United States	59.5 (40–75)	MTX plus UDCA/UDCA/OBS	UDCA: 13–15mg/kg/day MTX: 0.25mg/kg/week	2 years	121	NR/NR/NR	22/NR/NR
Leung (2011)	United States	54 (52–56)	MTX plus UDCA/COC plus UDCA	UDCA:13–15mg/kg/day; MTX:0.25mg/kg/week; COC: 1 mg/day	10 years	29	NR/NR	NR/NR
Battezzati (1993)	Italy	57 (42–60)	UDCA/OBS	500 mg per day	1 year	88	43/43	9/2
Combes (1995)	United States	49 (52–56)	UDCA/OBS	10 to 12 mg/kg/day	2 years	151	70/70	17/16
Eriksson (1997)	Sweden	57 (46–70)	UDCA/OBS	500 mg per day	2 years	116	58/52	12/7
Heathcote (1994)	Canada	56 (50–62)	UDCA/OBS	14mg/kg/day	2 years	222	91/94	16/24
Hwang (1993)	China	58 (50–66)	UDCA/OBS	600 mg/day.	3 months	12	6/6	17/17
Leuschner (1989)	German	53 (53–73)	UDCA/OBS	10 mg/kg/day	9 months	20	9/9	10/10
Lindor (1994)	United States	53 (51–55)	UDCA/OBS	13 to 15mg/kg/day	4 years	180	75/68	8/13
Oka (1990)	Japan	59 (51–67)	UDCA/OBS	600 mg/day	24 weeks	52	25/25	15/4
Papatheodoridis (2002)	Greece	54 (44–64)	UDCA/OBS	12 to 15 mg/kg/day	92 months	86	26/29	53/40
Pares (2000)	Spain	54 (44–64)	UDCA/OBS	14 to 16 mg/kg/day	2 years	192	89/89	26/18
Poupon (1991)	France	56 (47–65)	UDCA/OBS	13 to 15 mg/kg/day	2 years	146	67/63	15/26
Turner (1994)	England	57 (54–60)	UDCA/OBS	10mg/kg/day	2 years	46	21/21	27/29
Vuoristo (1995)	Finland	55 (50–60)	UDCA/OBS	12 to 15 mg/kg/day	2 years	59	29/26	3/29
Chazouilleres (1998)	France	50 (47–53)	UDCA/COT plus UDCA	UDCA: 13 to 15 mg/kg/day; COT: 0.5 mg/kg/day	23 months	11	3/5	20/33
Gunsar (2002)	Turkey	44 (40–55)	UDCA/COT plus UDCA	UDCA: 13 mg/kg/day; COT: 0.5 mg/kg/day	28 months	20	12/5	15/14
Chazouilleres (2006)	France	41 (39–57)	UDCA/COT plus UDCA	UDCA: 13 to 15 mg/kg/day; COT: 0.5 mg/kg/day	90 months	17	NR/NR	NR/NR
Heurgue (2007)	France	44 (41–56)	UDCA/COT plus UDCA	UDCA: 13 to 15 mg/kg/day; COT: 0.5 mg/kg/day	60 months	13	NR/NR	NR/NR
Ozaslan (2010)	Turkey	44 (40–51)	UDCA/COT plus UDCA	UDCA: 13 to 15 mg/kg/day; COT: 0.5 mg/kg/day	31 months	12	2/6	NR/NR
Tanaka (2011)	Japan	54 (50–60)	UDCA/COT plus UDCA	UDCA: 10 mg/kg/day; COT: 0.5 mg/kg/day	73 months	25	14/8	NR/NR
Ikeda (1996)	Japan	57 (55–60)	COC plus UDCA/UDCA	COC: 1 mg/kg/day; UDCA: 600 mg/day	30 months	22	NR/NR	30/8
Poupon (1996)	France	53 (51–55)	COC plus UDCA/UDCA	COC: 1 mg/d, 5 days per week; UDCA: 13–15 mg/day	2 years	74	NR/NR	5/3
Almasio (2000)	Italy	55 (45–65)	COC plus UDCA/UDCA	COC: 1 mg/daily; UDCA: 500 mg/daily	3 years	90	35/41	4/2
Battezzati (2001)	Italy	58 (48–68)	COC plus UDCA/UDCA	COC: 1 mg/daily; UDCA: 500 mg/daily	10 years	44	14/8	NR/NR
Christensen(1985)	Denmark	55 (25–78)	AZP/OBS	300 to 700 mg/week	11 years	248	58/72	6/7
Heathcote(1976)	UK	51 (24–79)	AZP/OBS	2 mg/kg/day	5 years	45	36/52	16/0
Lombard(1993)	UK	54 (44–64)	CYP/OBS	3 mg/kg/day	928 days	349	17/18	56/42
Minuk(1988)	Canada	51 (41–61)	CYP/OBS	2.5 mg/kg/day	1year	12	0/17	100/17
Wiesner(1990)	USA	46 (37–57)	CYP/OBS	4 mg/kg/day	2.7 years	29	5/30	79/50
Dickson(1985)	USA	46 (36–56)	DPM/OBS	250 mg/day	10 years	227	40/40	53/22
Epstein(1981)	UK	53 (43–63)	DPM/OBS	over 8 to 10 weeks from 150 mg/day to 600 mg/day	6 years	98	30/43	31/3
Matloff(1982)	USA	52 (42–62)	DPM/OBS	1g/day	28 months	52	62/12	35/4
Neuberger(1985)	UK	53 (41–62)	DPM/OBS	1.2 g/day, increased from 300 mg by 300 mg each fortnight until 1.2 g	4 years	189	54/32	36/8
Taal(1983)	Netherlands	51 (43–62)	DPM/OBS	1 g/day (increased from 250 mg every month until 1 g for the first 6 months. After that, decreased to 500 mg/day for the remaining 6 months	1 years	24	18/31	100/85
Mitchison (1992)	England	52 (44–60)	COT/OBS	initially 30 mg/day then reduced by 5 mg/day every two weeks until a maintenance dose of 10 mg/day was reached.	3 years	36	16/29	79/24
Hendrickse(1999)	England	57 (55–59)	MTX/OBS	7.5 mg/week.	6 years	60	40/37	83/73
Kaplan(2003)	USA	51 (50–53)	MTX plus COC	MTX: 15 mg/week, 5 mg every 12 hours 3 times.COC: 0.6 mg twice daily	2 years	85	57/44	17/7
Kaplan(1999)	USA	51 (50–53)	COC plus MTX	COC: 0.6 mg twice dailyMTX: 15 mg/wk, taken as 5 mg every 12 hours 3 times	24 months	85	23/29	NR/NR
Kaplan(1986)	USA	48 (40–68)	COC/OBS	0.6 mg twice daily.	2 years	60	20/47	13/3
Warnes(1987)	UK	53 (47–64)	COC/OBS	1 mg/day	18 months	64	15/30	56/7
Bodenheimer(1988)	USA	52 (34–59)	COC/OBS	0.6 mg twice daily.	5 years	57	NR/NR	14/3
Zifroni (1991)	USA	57 (50–65)	COC/OBS	0.6 mg twice daily.	4 years	57	46/48	11/0

Notes: COC: colchicine; BEF: bezafibrate; COT: corticosteroids; MTX: methotrexate; UDCA: ursodeoxycholic acid; OBS: observation; CYP: cyclosporin A; DPM: D-penicillamine; AZP: azathioprine; NR: not reported.

### Results from pair-wise comparisons

Seven different comparisons were accomplished in pairwise meta-analysis. The weighted HRs for MOLT and ORs for AEs, respectively, were calculated for each comparison. The geometric distribution of controlled trials on MOLT (Figure 1A) and AEs (Figure 1B) were displayed. The weighted hazard ratios and odds ratios for the two outcomes, MOLT and AEs, were calculated for each comparison. Statistical heterogeneity was assessed using the I^2^ statistic, which was assessable in two of the comparisons. Overall, statistical heterogeneity was moderate, although for some comparisons 95% CIs were wide and included values indicating very high or no heterogeneity (Table 2). In the meta-analyses of direct comparisons for efficacy, I² values higher than 75% were recorded for only two comparisons: COC plus UDCA *versus* UDCA (86.9%), with two studies in the meta-analysis, and DPM *versus* OBS, with five studies included. While in terms of AEs, I² values higher than 50% were recorded for only one comparison: CYP *versus* OBS (52.6%), with three studies in the meta-analysis, and the remaining comparisons were all lower than 40%. In addition, all *P* values for Begg's rank correlation test and Egger's test were greater than 0.05, indicating that no publication bias was found amongst those pairwise comparisons of different treatment regimens. (Table 3).

**Table 2:** Comparison of outcomes between pair-wise meta-analysis and network meta-analysis

Treatment comparisons	Results of pair-wise meta-analysis	Results of network meta-analysis
Clinical improvement		
MTX vs OBS	1.15 (0.41, 3.26)	0.95 (0.32, 2.78)
DPM vs OBS	1.51 (0.63, 3.61)	1.54 (0.76, 3.17)
CYP vs OBS	0.57 (0.17, 1.88)	0.53 (0.16, 1.46)
COT vs OBS	0.45 (0.09, 2.26)	0.43 (0.05, 3.34)
COC vs OBS	0.50 (0.24, 1.01)	0.57 (0.25, 1.26)
MTX vs COC	1.51 (0.80, 2.88)	1.67 (0.64, 4.29)
AZP vs OBS	0.54 (0.33, 0.88)	0.53 (0.18, 1.56)
UDCA vs OBS	0.93 (0.65, 1.31)	0.78 (0.45, 1.27)
UDCA vs UDCA plus COT	0.68 (0.20, 2.27)	0.38 (0.09, 1.39)
UDCA vs UDCA plus BEF	0.75 (0.04, 14.58)	0.77 (0.06, 9.89)
MTX plus UDCA vs UDCA	0.42 (0.03, 5.30)	0.74 (0.24, 2.44)
COC plus UDCA vs UDCA	1.17 (0.09, 14.55)	1.05 (0.33, 3.26)
Adverse events		
MTX VS OBS	1.82 (0.52, 6.38)	5.31 (1.21, 24.83)
COC vs MTX	2.67 (0.64, 11.11)	1.07 (0.23, 4.65)
DPM VS OBS	5.27 (3.34, 8.30)	8.00 (3.50, 22.46)
CYP VS OBS	3.24 (0.90, 11.64)	3.24 (1.21, 13.42)
COT VS OBS	12.19 (2.53, 58.72)	6.08 (0.80, 47.58)
COC VS OBS	8.91 (3.11, 25.56)	5.60 (1.95, 18.04)
AZP VS OBS	1.78 (0.25, 12.56)	1.60 (0.42, 7.30)
UDCA vs OBS	0.96 (0.64, 1.44)	0.95 (0.59, 1.56)
UDCA vs COT plus UDCA	1.08 (0.24, 4.89)	1.42 (0.17, 11.86)
UDCA vs UDCA plus BEF	2.58 (0.58, 11.57)	3.16 (0.59, 20.67)
UDCA vs UDCA plus MTX	1.30 (0.58, 2.94)	1.54 (0.50, 5.96)
UDCA vs UDCA plus COC	2.66 (0.65, 10.92)	3.12 (0.63, 18.75)

Notes: COC: colchicine; BEF: bezafibrate; COT: corticosteroids; MTX: methotrexate; UDCA: ursodeoxycholic acid; OBS: observation; CYP: cyclosporin A; DPM: D-penicillamine; AZP: azathioprine; NA: not available;

**Table 3:** Assessment of heterogeneity and publication bias for trials included in the traditional meta-analysis

Treatment comparisons	I^2^ (%)	P values of Begg's test	P values of Egger's test
Mortality or liver transplantation			
MTX vs OBS	NR	NR	NR
DPM vs OBS	79.8	0.62	0.84
CYP vs OBS	29.5	0.60	0.28
COT vs OBS	NR	NR	NR
COC vs OBS	16.5	0.60	0.44
MTX vs COC	0.0	0.32	NR
AZP vs OBS	0.0	0.32	NR
UDCA vs OBS	1.0	0.89	0.53
UDCA vs UDCA plus COT	0.0	0.33	0.08
UDCA vs UDCA plus BEF	0.0	NA	NA
MTX plus UDCA vs UDCA	0.0	NA	NA
COC plus UDCA vs UDCA	86.9	0.32	NA
Adverse events			
UDCA vs OBS	35.8	0.90	0.77
MTX vs OBS	NR	NR	NR
DPM vs OBS	0.0	0.62	0.13
CYP vs OBS	52.6	0.12	0.39
COT vs OBS	NR	NR	NR
COC vs OBS	0.0	1.00	0.23
MTX vs COC	NR	NR	NR
AZP vs OBS	45.7	0.32	NR
UDCA vs COT plus UDCA	0.0	0.12	0.06
UDCA vs UDCA plus BEF	0.0	0.12	0.12
UDCA vs UDCA plus MTX	3.3	0.32	NA
UDCA vs UDCA plus COC	0.0	0.12	0.30

Notes: COC: colchicine; BEF: bezafibrate; COT: corticosteroids; MTX: methotrexate; UDCA: ursodeoxycholic acid; OBS: observation; CYP: cyclosporin A; DPM: D-penicillamine; AZP: azathioprine; NA: not available.

### Results from the network meta-analysis of primary and secondary outcomes

**Figure 2:**
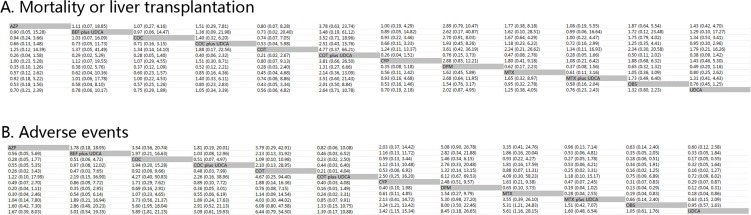
Clinical efficacy and safety of all treatments according to network meta-analysis Treatments are reported in alphabetical order. The ORs were estimated in upper and lower triangle comparing column-defining with row-defining treatment. **A.** Mortality or liver transplantation; **B.** Adverse events. For mortality or liver transplantation, HRs higher than 1 favor the column-defining treatment, while for adverse effects, ORs lower than 1 favor the row-defining treatment. COC: colchicine; BEF: bezafibrate; COT: corticosteroids; MTX: methotrexate; UDCA: ursodeoxycholic acid; CYP: cyclosporin A; DPM: D-penicillamine; AZP: azathioprine; OBS: observation. A. Mortality or liver transplantation; B. Adverse events.

Figure 2A and Figure 2B illustrates the HRs and ORs for MOLT and AEs respectively and 95% confidence intervals obtained from the indirect comparisons of the included regimens. Following Figure 2A from left to right, although differing not significantly, there was a trend that combination of COT plus UDCA was most effective in reducing the risk of MOLT than AZP (HR 3.78, 95%CI 0.63 to 23.74), COC (HR 3.52, 95%CI 0.71 to 18.96), COT (HR 4.77, 95%CI 0.37 to 66.21), CYP (HR 3.81, 95%CI 0.66 to 26.50), DPM (HR 1.31, 95%CI 0.27 to 6.66), MTX (HR 2.14, 95%CI 0.36 to 13.09), OBS (HR 2.01, 95%CI 0.50 to 8.84), monotherapy with UDCA (HR 2.63, 95%CI 0.72 to 10.75), COC plus UDCA (HR 2.55, 95%CI 0.44 to 15.03), MTX plus UDCA (HR 3.58, 95%CI 0.61 to 21.96) or BEF plus UDCA (HR 3.48, 95%CI 0.21 to 56.93). Consistently, Figure 2B shows that for AEs, when compared with OBS, COC (HR 0.18, 95%CI 0.06 to 0.51), CYP (HR 0.31, 95%CI 0.07 to 0.83), DPM (HR 0.13, 95%CI 0.04 to 0.29) and MTX (HR 0.19, 95%CI 0.04 to 0.83) yielded a significant difference in causing AEs. In addition, compared with UDCA, a statistical significance was also achieved in COC (HR 0.17, 95%CI 0.05 to 0.55), CYP (HR 0.29, 95%CI 0.07 to 0.87), DPM (HR 0.12, 95%CI 0.04 to 0.31) and MTX (HR 0.18, 95%CI 0.04 to 0.86).

**Figure 3:**
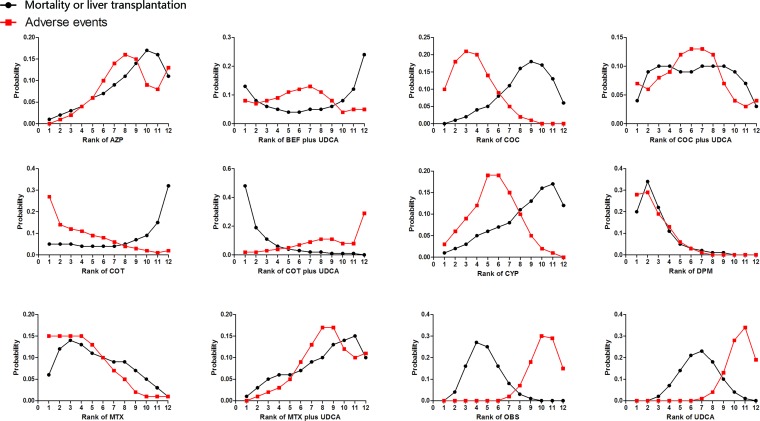
Rankograms showing probability of each strategy having each specific rank (1-6) for mortality or liver transplantation and adverse events Ranking indicates the probability to be the best treatment, the second best, the third best and so on. Rank 1 is worst and rank N is best. COC: colchicine; BEF: bezafibrate; COT: corticosteroids; MTX: methotrexate; UDCA: ursodeoxycholic acid; CYP: cyclosporin A; DPM: D-penicillamine; AZP: azathioprine; OBS: observation.

**Figure 4:**
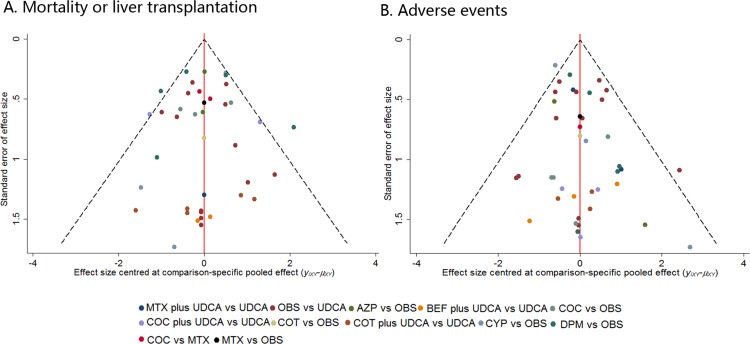
Comparison-adjusted funnel plot for the treatment network in terms of mortality or liver transplantation and adverse events The red line represents the null hypothesis that the study-specific effect sizes do not differ from the respective comparison-specific pooled effect estimates. Different colors correspond to different comparisons. Estimates below one indicate that the benefit of the experimental intervention is more pronounced in the trial than the pooled estimate. Observations from small studies missing on the right side of the line of null effect (ratio of rate ratios > 1) indicate that small studies tend to exaggerate the effectiveness of experimental treatments. COC: colchicine; BEF: bezafibrate; COT: corticosteroids; MTX: methotrexate; UDCA: ursodeoxycholic acid; CYP: cyclosporin A; DPM: D-penicillamine; AZP: azathioprine; OBS: observation. **A.** Mortality or liver transplantation; **B.** Adverse events.

Figure 3 shows the distribution of probabilities for each treatment being ranked at different positions for the outcome of MOLT or AEs. Although the efficacy effects of combined therapy of COT plus UDCA compared with OBS compared with placebo was not statistically significant, COT plus UDCA had the greatest probability (48%) for being the best treatment option on reducing risk of MOLT. While in terms of AEs, DPM had the highest probabilities (28%) of reduction in AEs (Figure 3), suggesting DPM was more likely to cause AEs than remaining treatments. On the other hand, COC or COT plus UDCA demonstrated the least adverse events, the cumulative probabilities of being ranked first and second in with respect to the safety profile was combination of COC or COT and UDCA. Figure 4 presents comparison-adjusted funnel plot for UDCA-based therapies network, without the evidence of asymmetry.

### Comparisons between traditional pairwise and network meta-analyses

Table 2 shows the results of traditional pairwise and network meta-analyses. Although the pooled estimates showed small differences, the confidence intervals from traditional pairwise meta-analyses and the credible intervals from Bayesian network meta-analyses in general overlapped. The P-values show no significant difference between the direct and indirect effects (Table 4). In general, the node splitting method showed no significant inconsistency within the networks for any of the two outcomes.

**Table 4:** Assessment of inconsistency between direct and indirect evidence

Treatment comparisons	P value of node-splitting method
Mortality or liver transplantation	
COC vs MTX	0.76
COC vs OBS	0.21
COC vs UDCA	0.10
MTX vs OBS	0.73
COC plus UDCA vs MTX plus UDCA	0.69
COC plus UDCA vs UDCA	0.67
MTX plus UDCA vs UDCA	0.69
Adverse events	
COC vs MTX	0.07
COC vs OBS	0.07
MTX vs OBS	0.07

Notes: COC: colchicine; MTX: methotrexate; UDCA: ursodeoxycholic acid; OBS: observation

### Sensitivity analysis

In the sensitivity analysis, there were eleven trials [2, 12, 22, 24–25, 27–28, 31, 33, 35, 39] excluded which reported patients administrated by high (more than 500 mg/kg/day) dose of UDCA (BEF plus UDCA excluded). Thirty-eight independent studies were performed for the primary outcome, MOLT. Overall, the sensitivity analysis showed that omitting those trials with high dose UDCA did not impact on the ranking order and statistical significance of the remaining treatments. Supplementary 3A indicates that combination of COT and UDCA was the top-ranked treatments, although differ not significantly. Similar findings were also observed for the outcome of AEs. DPM, COC or CYP were associated with significant effects in causing AEs compared with OBS. Besides, COT or COC plus UDCA were ranked the least possibility to cause AEs (Supplementary 3B).

## DISCUSSION

In this network meta-analysis reviewing the efficacy and safety of most comprehensive interventions in patients with PBC to date, we found that combined therapy with COT and UDCA was the most effective in reducing the risk of MOLT with a weighted benefit-risk ratio for patients with PBC. Monotherapy with DPM, CYP, AZP or MTX received statistical significance in causing AEs where treatment with DPM showed higher probabilities of being at the superior ranking positions in AEs. These estimates are fairly robust and changed little in sensitivity analyses.

Our study has several strengths. Firstly, our network meta-analysis, until more evidences of direct active comparisons are reported, provides a useful and complete picture for propensity of most comprehensive treatments associated with efficacy and safety outcomes among patients with PBC. Network meta-analysis allowed comparison of all available strategies in a single analysis to give a combined total 4182 patients of treatments, rather than separate and disconnected meta-analyses for individual pairs of treatments. Secondly, we were able to provide a formal rank order for all treatment strategies by their capacity to reduce the risk of MOLT or AEs by conducting a network meta-analysis, and this is only attainable by using a Bayesian approach. Finally, in order to reduce concerns on potential inconsistency, we performed inconsistency diagnostic for all triangular and quadrilateral loops. In addition, statistical heterogeneity was moderate, although for some I² values of comparisons, heterogeneity was high (Table 2). In order to test the robustness of our results due to high heterogeneity values in some comparisons, we performed sensitivity analysis regarding to patients administrated by high (more than 500 mg/kg/day) dose of UDCA (BEF plus UDCA excluded), which showed that the results was similar to our main analysis. Furthermore, we analyzed the effects of AEs to obtain a favorable benefit-risk ratio for patients with PBC based on all treatments.

We do also have to acknowledge several limitations in the analysis. Firstly, it may be argued that the inclusion of trial patients with optimum dose of UDCA and trials with high dose may have biased results. However, a sensitivity analysis excluding trials in trials where patients were administrated by high dose of UDCA yielded much the same results as the primary analysis. Secondly, the study size assigned to different comparisons was overall small in most included studies in this analysis. However, our study had established the largest PBC treatment sample size for trials performed to date in the world. Thirdly, Factors such as trial heterogeneity, bias and inconsistency can affect the estimates reported in the study [56]. For instance, this analysis was performed on the assumption of consistency where the validity of indirect comparisons was determined by the extent of clinical and methodological trial similarity. However, inconsistency remains a methodological issue of multiple treatment comparisons, as it arises from pooling the data and small number of trials available for the different comparisons resulting in discrepancies between the direct and indirect comparisons, and consequently threatening the validity of the results [56–58]. Furthermore, we are unable to provide comparisons of drug efficacy based on disease duration, MELD, and the presence of cirrhosis due to lack of the above information reported from included trials. Hence, further studies need to be conducted on disease duration, MELD, and the presence of cirrhosis. In the case of our network meta-analysis, the indirect estimates were often very similar to those obtained in the direct comparisons because only single comparisons were available for the majority of the cases. This resulted in a less conventional geometry where our network of trials did not have any closed loops. Finally, our analysis was that not all AE outcomes of interest were reported consistently across trials. Furthermore, there were cases where no events had occurred for the outcome of interest resulting in the requirement to add a continuity correction to the results [58]. In general, missing data resulted in wider credible intervals due to greater uncertainty around the estimates. However, despite these limitations, this network meta-analysis provides the largest scale comparative information on the major clinical outcome profiles of different interventions in current use.

Our study results are consistent with some of previous pairwise meta-analyses, but the network meta-analysis incorporates both direct and indirect comparisons of treatment strategies, including those that have never been compared directly. UDCA appears a safe regimen and may be useful for preventing the progression of PBC, which is the only therapy approved by the U.S. Food and Drug Administration [59]. However, one traditional meta-analysis in 2008 [10] and another published in 2012 [11] of 16 RCTs both reported that, although differing not significantly, they did not demonstrate any benefit of UDCA on mortality and MOLT of patients with PBC. These findings are consistent with our results. In addition, some trials have also concluded that UDCA has no beneficial effect on patient survival, but may be a safe option for patients with PBC. As previously reported in a Canadian Multicenter RCT (reference here), UDCA, when administered to patients with PBC, led to an improvement in clinical outcomes. However, a larger data sample is needed to determine whether UDCA therapy has a beneficial effect on PBC patient survival. Hence, one possible explanation for seemingly inconsistent results, was that the small patient population in those trials which supported UDCA was beneficial to patients.

Although our analysis did not demonstrate that combination therapy of COT with UDCA had a significant risk reduction in MOLT, we found the evidence favoring COT plus UDCA compared with OBS (HR 2.01, 95%CI 0.5 to 8.84), and further network meta-analysis results also revealed similar non-significant relationship. The results of our analysis on efficacy are remarkably similar with several studies. A previous meta-analysis in 2013 [60] was performed of RCTs concluded that the combination therapy of UDCA and COT was more effective in comparison to monotherapy with UDCA for patients with PBC, which we also found in both our direct and indirect comparisons. Similarly, two RCTs [61–62] of the analysis showed that COT plus UDCA appeared to be the best therapeutic option for PBC patients. In addition, COT plus UDCA had the probability of being ranked second with respect to safety outcome. Few AEs were reported, which included osteoporosis, bleeding, aggravated itching, and diarrhea in only two included RCTs [26, 29] associated with COT plus UDCA.

Besides, other single therapies, such as DPM, CYP, AZP or MTX, were also evaluated by several studies. The results of our analysis showed compared with OBS, CYP, DPM or MTX were significantly more likely to cause AEs. Further, no survival benefits can also be found in treatments with CYP, AZP, MTX or MTX. As reported by AASLD in 2009 [59], the clinical guidelines do not recommend any of above treatments. Some head-to-head meta-analyses [63–65] all found that treatments with AZP, CYP or MTX did not appear to reduce the risk of MOLT, and led to more AEs for patients with PBC, which are consistent with our results. As is showed in our analysis, treatment with DPM had the highest probabilities of being ranked top in adverse-effects, which included rash, thrombocytopenia, myasthenia, arthralgia, etc [42–43, 48, 52]. In addition, two following traditional meta-analyses [66–67] showed that treatment with DPM was associated with little clinical benefits over OBS, and increased the risk of causing the AEs. Therefore, based on our analysis incorporating direct and indirect evidences, there was little evidence favoring the use of these single drugs in clinical efficacy or safety profile.

In conclusion, the study suggests that the superiority of using COT plus UDCA in the treatment of PBC with weighted benefit-risk ratio in MOLT and AEs. For outcome of AEs, UDCA-based therapies, such as UDCA, COT plus UDCA or COC plus UDCA, all showed well-tolerated in adverse effects. While single treatments with CYP, DPM or MTX all have achieved statistically significant as compared with OBS, increased risk of AEs where DPM was most likely to cause AEs.

## MATERIALS AND METHODS

### Search strategy

We adhered to the PRISMA (Preferred Reporting Items for Systematic reviews and Meta-Analyses) statement for reporting systematic reviews and meta-analyses in healthcare interventions (Supplementary 4) [68]. Four electronic databases (PubMed, Scopus, Embase, and the Cochrane Library) were searched until the end of August 2014 for randomized controlled trials investigating any treatments for patients with PBC, with the key terms “treatments/therapies, primary biliary cirrhosis, randomized clinical trial” without any language or date restrictions. We also searched the additional studies in the reference lists of all identified publications, including relevant meta-analyses and systematic reviews. Two reviewers (Zhu GQ, Huang S) independently assessed the eligibility of all potential abstracts and titles. Any disagreements were resolved through discussion and repeat extraction by a third reviewer (Zheng MH).

### Selection criteria

We included randomized, placebo, or untreated controlled clinical trials comparing the effects of any single or combination of treatments with observation or other classes of active treatments in patients with PBC older than 18 years old. Included studies had to report at least one of two outcomes: MOLT and adverse events (AEs). PBC was diagnosed according to established diagnostic criteria (at least three of the following criteria: alkaline phosphatase and (or gamma glutamyl transpeptidase above the upper limit of normal; antimitochondrial antibodies positive at a titer of 1:40; absence of biliary obstruction by ultrasonography or other radiological tests; or compatible liver biopsy). Other exclusions were trials that comprised a non-randomized design, reviews or pooled-analyses and assessments of other therapies and studies with no usable outcomes data. Duplications were eliminated for the same title, author list or publication date. Eligible studies had to be published as full length articles.

### Data extraction

Two investigators (Zhu GQ, Shi KQ) independently extracted data from each study and enter it into a database. Any discrepancies regarding the extraction of data were resolved by additional investigator (Zheng MH). Data were extracted from each study with a predesigned electronic database, including publication data (the first author's name, year of publication, and country of population studied), treatment protocols that were compared and number of patients assigned to each group, the number of events of interest in each group. When relevant information on design or outcomes was unclear, or when doubt existed about duplicate publications, we contacted the original authors for clarification. We choose two clinically meaningful events to estimate efficacy and safety separately of treatments for network meta-analysis: MOLT, which was commonly considered as the most important evaluation, and AEs, including serious events (defined as any untoward medical occurrence that was life threatening, resulted in death, or was persistent or led to significant disability) or non-serious events (that is, any medical occurrence not necessarily having a causal relationship with the treatment). When relevant information on design or outcomes was unclear, or when some needed data was unavailable directly from the study, the original authors were contacted for clarifications and assistance by email.

### Study quality

Two independent reviewers assessed the quality of the methodology by using the Cochrane Risk of Bias Tool, an established tool based on assessing sequence generation for the randomization of subjects, allocation concealment of treatment, blinding, incomplete outcome data, selective outcome reporting and other sources of bias [69]. Trials with high or unclear risk for bias for any one of the first three components were regarded as trials with high risk of bias. Otherwise, they were considered as trials with low risk of bias.

### Data analysis

All data from each eligible study were extracted and entered into a standardized spreadsheet (Microsoft Excel 2007; Microsoft, Redmond, WA). We analyzed two treatment outcomes separately (MOLT, AE). Firstly, traditional pairwise meta-analyses were conducted for studies that directly compared different treatment arms. Then we performed Bayesian network meta-analyses to compare different PBC therapies.

Firstly, we performed traditional pairwise meta-analyses for studies that directly compared different treatment arms with STATA 12.0 (Stata Corporation, College Station, Texas, USA). We calculated, by using the method of DerSimonian and Laird random effects model, of hazards ratios, the pooled estimates of odds ratios and 95% confidence intervals of direct comparisons between two strategies according to Cochrane Handbook for Systematic Reviews of Interventions Version 5.1.0. Clinical heterogeneity was first assessed through clinical judgment with input from experts in the field. Statistical heterogeneity was then assessed by the *P* values of Egger's and Begg's test from pair-wise meta-analysis, which was higher than 0.05 suggesting heterogeneity. A formal assessment of heterogeneity was then accomplished by referring to the I^2^ statistic. According to the standard guidelines, I^2^ values greater than 50% are considered high heterogeneity levels, between 25-50% moderate and less than 25% considered low heterogeneity levels. Once the heterogeneity was suspected, sensitivity analysis was employed [70]. It was conducted based on the important covariate, the dose of UDCA, to investigate the robustness of our main analyses. The sensitivity analysis for dose of UDCA included trials that patients administrated by a dose of 13-15 mg/kg/day, according to AASLD practice guidelines in 2009 [59].

In addition to the direct comparison meta-analyses, we performed multiple-treatment meta-analysis with a random-effects model within a Bayesian framework using Markov chain Monte Carlo methods in WinBUGS (MRC Bio- statistics Unit, Cambridge, UK). The advantages of using a bayesian analytical approach are that direct probability statements on treatment comparisons can be made and that all evidence for a specific problem can be taken into account as it includes evidence on both indirect and direct comparisons and as such allows estimation of the comparisons between interventions that have not been examined directly in previous trials [16, 18]. The pooled estimates were obtained using the Markov chains Monte Carlo method. As we have described in our previous published network meta-analysis [2, 26, 30, 39], analyses were based on non-informative priors for relative-effect parameters (flat normal with mean of 0 and precision of 0.001) and between-study SD (a flat uniform distribution between 0 and 2). Convergence and lack of autocorrelation were checked and confirmed after a 5000-simulation burn-in phase without any thinning and using 4 chains with different initial values. Then, a burn-in phase of 20 000 iterations was used, followed by 50 000 iterations to estimate parameters. This method combined direct and indirect evidence for any given pair of treatments in one joint analysis [71–72].

We compared the pooled HRs from the network meta-analysis with corresponding HRs from pair-wise random-effects meta-analysis of direct comparisons to assess whether there was inconsistency between direct and indirect comparisons. Odds ratios (ORs) were calculated from the number of total patients and the number of patients in each trial for network meta-analysis for AEs. In addition, to estimate inconsistency, we used the node splitting method to calculate the inconsistency of the model, which separated evidence on a particular comparison into direct and indirect evidence [72]. Bayesian *P* value was also reported and evaluated between the direct and indirect evidence [72].

The treatments were ranked for each outcome in each simulation on the basis of their posterior probabilities. We assessed the probability that each treatment was the most efficacious regimen, the second best, the third best and so on, by calculating the HR for each treatment compared with an arbitrary common group and counting the proportion of iterations of the Markov chain in which each treatment had the highest HR, the second highest, and so on. Even though the differences in effect size among treatments may be small, clinical decisions about the choice of treatments can still be suggested based on the probabilities of treatment ranking [26, 73]. The outcome of AEs was also reported as odds ratios with corresponding 95% credible intervals, as well as the probabilities of ranking by treatment. Therefore, the network meta-analysis increased statistical power by incorporating evidence from both direct and indirect comparisons across all treatments.

## SUPPLEMENTARY MATERIAL


