# Supplementary material for: Optimal drug regimens for primary biliary cirrhosis: a systematic review and network meta-analysis
Source: Oncotarget. 2015 Jun 19;6(27):24533–49. doi: 10.18632/oncotarget.4528 (PMC4695204; doi:10.18632/oncotarget.4528)
Supplement: Supplementary file 1 [file oncotarget-06-24533-s001.pdf]

Optimal drug regimens for primary biliary cirrhosis: a systematic review and network meta-analysis

Supplementary Material

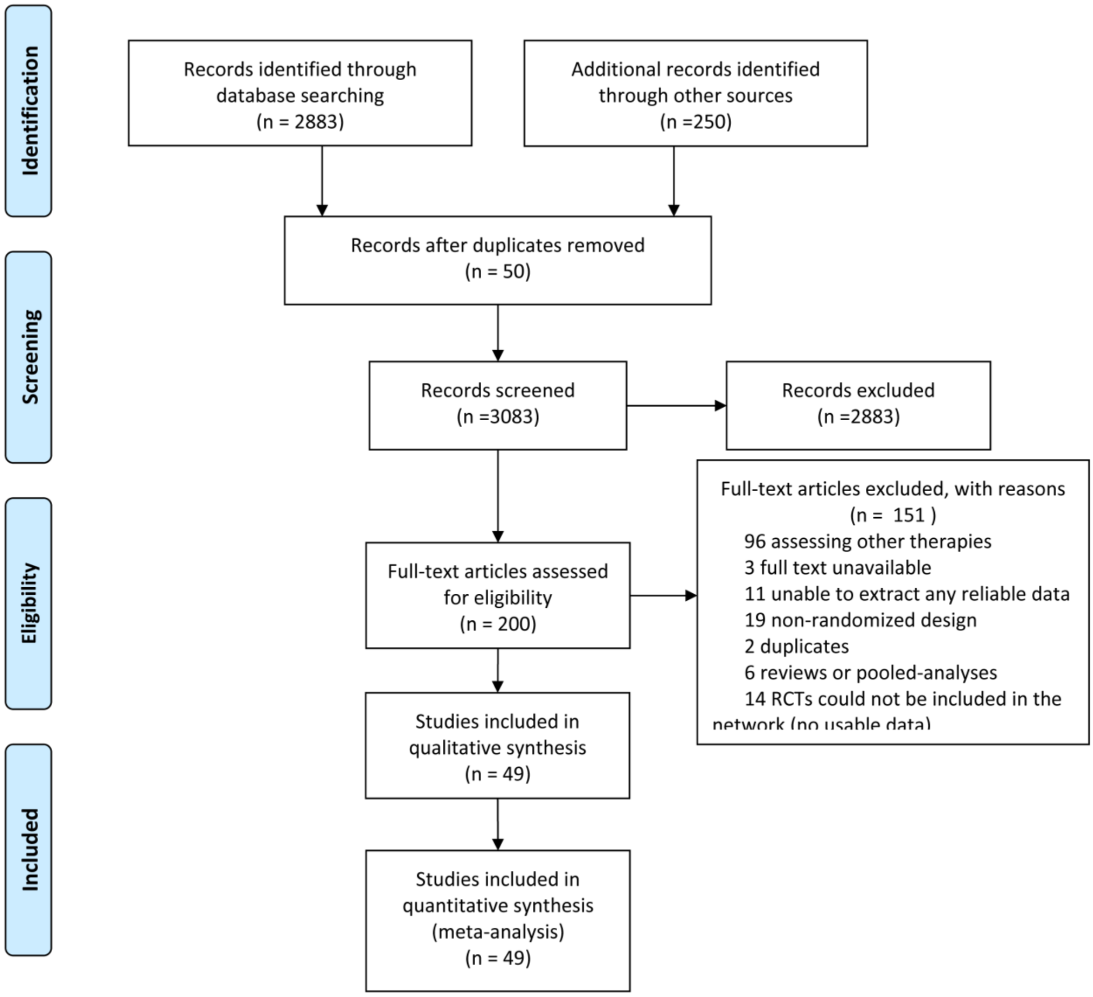

Supplementary 1. PRISMA Flow Diagram

	Random sequence generation (selection bias)	Allocation concealment (selection bias)	Blinding of participants and personnel (performance bias)	Blinding of outcome assessment (detection bias)	Incomplete outcome data (attrition bias)	Selective reporting (reporting bias)	Other bias
Almasio 2000	+	?	+	+	−	+	+
Battezzati 1993	+	+	+	+	+	+	?
Battezzati 2001	+	+	+	+	+	?	+
Bodenheimer 1988	+	?	+	+	+	+	?
Chazouillères 1998	+	?	?	?	+	+	?
Chazouilleres 2006	−	−	?	?	+	+	+
Chiristensen 1985	+	+	+	+	+	?	?
Combes 1995	?	?	+	+	+	+	+
Combes 2005	?	?	?	?	+	−	−
Dickson 1985	+	+	+	+	+	+	+
Epstein 1981	?	?	+	+	?	+	?
Eriksson 1997	+	?	+	+	+	+	+
Gonzalez-Koch 1997	?	?	?	?	+	+	−
Günsar 2002	?	?	?	?	+	+	+
Heathcote 1976	?	+	−	−	+	+	+
Heathcote 1994	+	+	+	+	+	+	+
Hendrickse 1999	+	+	+	+	?	+	?
Henry 1988	+	?	+	+	+	+	+
Heurgue 2007	−	−	?	?	+	+	+
Hwang 1993	?	?	+	+	+	+	+
Ikeda 1996	+	?	+	+	+	+	+
Itakura 2004	?	?	?	?	+	+	+
Iwasaki 2008	+	+	−	−	+	+	+
Kanda 2003	?	?	?	?	+	+	−
Kaplan 1986	?	?	?	?	+	+	−
Kaplan 2003	+	+	+	+	+	+	+
Leung 2011	+	+	+	?	+	+	?
Leuschner 1989	?	?	+	+	+	+	+
Leuschner 1999	+	+	+	+	+	+	?
Lindor 1994	+	+	+	+	+	+	+
Lindor 1995	+	?	?	?	?	+	+
Lombard 1993	+	+	+	?	+	?	+
Matloff 1982	?	+	+	+	−	+	?
Minuk 1988	+	?	?	?	+	+	+
Mitchison 1992	+	+	+	+	?	+	?
Neuberger 1985	?	?	−	−	+	+	+
Oka 1990	?	+	+	+	+	+	+
Ozaslan 2010	?	?	?	?	+	+	+
Papatheodoridis 2002	+	+	?	?	+	+	?
Pares 2000	+	+	+	+	+	+	?
Poupan 1996	+	?	+	?	+	+	+
Poupon 1991	?	?	+	+	+	+	+
Taal 1983	?	?	+	+	+	?	+
Tanaka 2011	+	?	+	+	+	+	+
Turner 1994	?	?	+	+	+	+	?
Vuoristo 1995	?	?	?	?	+	+	?
Warnes 1987	+	+	+	?	+	?	+
Wiesner 1990	?	?	+	?	?	+	+
Zifroni 1991	+	?	+	?	+	+	+

Supplementary 2. Summary for risk of bias of included randomized controlled trials. The green symbols represent low risk of bias, the yellow symbols represent unclear risk of bias, and the red symbols represent high risk of bias. The figure was generated using Review Manager Version 5.

A. Mortality or liver transplantation

AZP	1.11 (0.02, 52.31)	1.05 (0.28, 3.91)	1.06 (0.05, 26.89)	0.78 (0.08, 7.40)	3.92 (0.65, 22.79)	1.03 (0.20, 4.31)	2.83 (0.83, 10.16)	1.77 (0.40, 7.52)	1.08 (0.16, 6.74)	1.86 (0.65, 5.43)	1.44 (0.44, 4.64)
0.90 (0.02, 53.33)	BEF plus UDCA	0.95 (0.02, 53.18)	0.98 (0.01, 115.05)	0.71 (0.01, 59.12)	3.55 (0.08, 210.56)	0.91 (0.02, 51.17)	2.61 (0.05, 145.58)	1.61 (0.03, 96.69)	0.98 (0.02, 59.24)	1.69 (0.04, 86.28)	1.31 (0.03, 62.15)
0.95 (0.26, 3.63)	1.05 (0.02, 47.11)	COC	0.99 (0.05, 22.50)	0.74 (0.08, 6.43)	3.71 (0.72, 19.56)	0.98 (0.24, 3.43)	2.69 (0.95, 7.74)	1.67 (0.66, 4.31)	1.01 (0.18, 5.77)	1.77 (0.80, 3.89)	1.38 (0.53, 3.37)
0.94 (0.04, 22.09)	1.02 (0.01, 106.23)	1.01 (0.04, 20.35)	COC plus UDCA	0.74 (0.02, 25.72)	3.83 (0.13, 94.10)	0.95 (0.04, 21.85)	2.66 (0.13, 57.58)	1.66 (0.07, 37.20)	1.05 (0.08, 12.13)	1.79 (0.09, 33.14)	1.38 (0.07, 23.98)
1.29 (0.14, 13.28)	1.42 (0.02, 99.35)	1.36 (0.16, 12.64)	1.35 (0.04, 50.91)	COT	5.03 (0.41, 65.76)	1.28 (0.12, 12.78)	3.63 (0.42, 33.13)	2.23 (0.24, 24.08)	1.34 (0.11, 18.54)	2.37 (0.32, 19.79)	1.81 (0.23, 16.07)
0.25 (0.04, 1.53)	0.28 (0.00, 12.92)	0.27 (0.05, 1.39)	0.26 (0.01, 7.60)	0.20 (0.02, 2.43)	COT plus UDCA	0.26 (0.04, 1.48)	0.73 (0.15, 3.59)	0.45 (0.07, 2.77)	0.27 (0.04, 1.93)	0.48 (0.11, 2.08)	0.37 (0.09, 1.39)
0.97 (0.23, 5.00)	1.10 (0.02, 56.82)	1.02 (0.29, 4.24)	1.05 (0.05, 27.09)	0.78 (0.08, 8.16)	3.85 (0.68, 25.63)	CYP	2.73 (0.86, 11.63)	1.72 (0.41, 8.74)	1.03 (0.17, 8.19)	1.80 (0.67, 6.11)	1.40 (0.46, 5.25)
0.35 (0.10, 1.20)	0.38 (0.01, 18.22)	0.37 (0.13, 1.05)	0.38 (0.02, 7.73)	0.28 (0.03, 2.37)	1.38 (0.28, 6.76)	0.37 (0.09, 1.17)	DPM	0.62 (0.17, 2.13)	0.38 (0.07, 2.09)	0.66 (0.33, 1.29)	0.51 (0.21, 1.18)
0.56 (0.13, 2.48)	0.62 (0.01, 29.20)	0.60 (0.23, 1.51)	0.60 (0.03, 14.27)	0.45 (0.04, 4.12)	2.22 (0.36, 13.50)	0.58 (0.11, 2.44)	1.60 (0.47, 5.88)	MTX	0.61 (0.09, 3.89)	1.05 (0.37, 3.07)	0.82 (0.25, 2.59)
0.92 (0.15, 6.19)	1.02 (0.02, 53.07)	0.99 (0.17, 5.57)	0.95 (0.08, 13.13)	0.75 (0.05, 9.38)	3.72 (0.52, 26.96)	0.97 (0.12, 5.89)	2.66 (0.48, 14.09)	1.64 (0.26, 10.62)	MTX plus UDCA	1.75 (0.37, 8.05)	1.36 (0.32, 5.67)
0.54 (0.18, 1.53)	0.59 (0.01, 24.12)	0.57 (0.26, 1.24)	0.56 (0.03, 11.62)	0.42 (0.05, 3.11)	2.09 (0.48, 9.15)	0.56 (0.16, 1.49)	1.52 (0.77, 3.07)	0.95 (0.33, 2.70)	0.57 (0.12, 2.71)	OBS	0.77 (0.45, 1.30)
0.69 (0.22, 2.27)	0.76 (0.02, 31.15)	0.72 (0.30, 1.88)	0.73 (0.04, 14.08)	0.55 (0.06, 4.36)	2.68 (0.72, 10.86)	0.71 (0.19, 2.18)	1.95 (0.85, 4.86)	1.22 (0.39, 4.05)	0.74 (0.18, 3.16)	1.29 (0.77, 2.21)	UDCA

B. Adverse events

AZP	3.40 (0.48, 22.39)	1.22 (0.04, 57.65)	3.84 (0.28, 49.58)	0.64 (0.04, 9.80)	2.10 (0.34, 17.44)	5.03 (0.85, 30.25)	3.19 (0.34, 26.03)	0.52 (0.04, 5.18)	0.61 (0.12, 2.57)	0.47 (0.08, 2.19)
0.29 (0.04, 2.10)	COC	0.35 (0.01, 16.05)	1.12 (0.10, 13.11)	0.18 (0.01, 2.47)	0.62 (0.13, 4.02)	1.46 (0.33, 7.28)	0.92 (0.19, 4.49)	0.15 (0.02, 1.26)	0.18 (0.05, 0.54)	0.14 (0.03, 0.48)
0.82 (0.02, 26.66)	2.90 (0.06, 78.43)	COC plus UDCA	3.09 (0.05, 140.51)	0.53 (0.01, 22.41)	1.77 (0.04, 58.47)	4.21 (0.10, 112.51)	2.66 (0.05, 84.65)	0.43 (0.01, 13.18)	0.51 (0.01, 11.20)	0.39 (0.01, 7.64)
0.26 (0.02, 3.54)	0.89 (0.08, 10.13)	0.32 (0.01, 19.46)	COT	0.16 (0.01, 3.85)	0.56 (0.05, 7.66)	1.30 (0.14, 14.76)	0.83 (0.06, 11.54)	0.13 (0.01, 2.05)	0.16 (0.02, 1.28)	0.12 (0.01, 1.07)
1.56 (0.10, 26.18)	5.46 (0.41, 71.34)	1.88 (0.04, 125.45)	6.14 (0.26, 137.63)	COT plus UDCA	3.38 (0.27, 57.07)	7.83 (0.67, 103.45)	5.03 (0.31, 78.13)	0.81 (0.05, 13.66)	0.96 (0.09, 9.46)	0.73 (0.08, 6.55)
0.48 (0.06, 2.91)	1.61 (0.25, 7.93)	0.57 (0.02, 23.28)	1.79 (0.13, 19.18)	0.30 (0.02, 3.75)	CYP	2.40 (0.45, 9.89)	1.52 (0.17, 9.45)	0.25 (0.02, 1.84)	0.29 (0.06, 0.83)	0.22 (0.04, 0.72)
0.20 (0.03, 1.18)	0.68 (0.14, 2.99)	0.24 (0.01, 9.78)	0.77 (0.07, 7.38)	0.13 (0.01, 1.48)	0.42 (0.10, 2.23)	DPM	0.64 (0.09, 3.76)	0.10 (0.01, 0.73)	0.12 (0.04, 0.29)	0.09 (0.02, 0.26)
0.31 (0.04, 2.95)	1.09 (0.22, 5.28)	0.38 (0.01, 19.72)	1.21 (0.09, 17.23)	0.20 (0.01, 3.28)	0.66 (0.11, 5.81)	1.57 (0.27, 10.78)	MTX	0.16 (0.01, 1.72)	0.19 (0.04, 0.90)	0.15 (0.03, 0.76)
1.93 (0.19, 23.51)	6.69 (0.79, 62.92)	2.33 (0.08, 134.33)	7.51 (0.49, 137.99)	1.23 (0.07, 22.13)	4.02 (0.54, 52.91)	9.66 (1.38, 93.69)	6.13 (0.58, 76.54)	MTX plus UDCA	1.17 (0.20, 7.86)	0.91 (0.16, 5.23)
1.64 (0.39, 8.00)	5.60 (1.85, 19.12)	1.96 (0.09, 76.60)	6.28 (0.78, 53.75)	1.04 (0.11, 10.98)	3.41 (1.21, 15.71)	8.17 (3.41, 24.94)	5.26 (1.11, 24.76)	0.85 (0.13, 5.07)	OBS	0.76 (0.39, 1.39)
2.13 (0.46, 12.26)	7.34 (2.09, 30.34)	2.56 (0.13, 96.32)	8.27 (0.93, 82.51)	1.37 (0.15, 13.29)	4.46 (1.39, 24.79)	10.62 (3.80, 41.26)	6.87 (1.32, 37.47)	1.10 (0.19, 6.27)	1.31 (0.72, 2.54)	UDCA

Supplementary 3. Clinical efficacy and safety of all treatments according to network meta-analysis in the sensitivity analysis

Section/Topic	#	Checklist Item	Reported on Page #
**TITLE**			
Title	1	Identify the report as a systematic review, meta-analysis, or both.	1
**ABSTRACT**			
Structured summary	2	Provide a structured summary including, as applicable: background; objectives; data sources; study eligibility criteria, participants, and interventions; study appraisal and synthesis methods; results; limitations; conclusions and implications of key findings; systematic review registration number.	1
**INTRODUCTION**			
Rationale	3	Describe the rationale for the review in the context of what is already known.	1-2
Objectives	4	Provide an explicit statement of questions being addressed with reference to participants, interventions, comparisons, outcomes, and study design (PICOS).	2
**METHODS**			
Protocol and registration	5	Indicate if a review protocol exists, if and where it can be accessed (e.g., Web address), and, if available, provide registration information including registration number.	
Eligibility criteria	6	Specify study characteristics (e.g., PICOS, length of follow-up) and report characteristics (e.g., years considered, language, publication status) used as criteria for eligibility, giving rationale.	11-13
Information sources	7	Describe all information sources (e.g., databases with dates of coverage, contact with study authors to identify additional studies) in the search and date last searched.	11
	8	Present full electronic search strategy for at least one database, including any limits used, such that it could be repeated.	11
	9	State the process for selecting studies (i.e., screening, eligibility, included in systematic review, and, if applicable, included in the meta-analysis).	11
Data collection process	10	Describe method of data extraction from reports (e.g., piloted forms, independently, in duplicate) and any processes for obtaining and confirming data from investigators.	12
Data items	11	List and define all variables for which data were sought (e.g., PICOS, funding sources) and any assumptions and simplifications made.	12
Risk of bias in individual studies	12	Describe methods used for assessing risk of bias of individual studies (including specification of whether this was done at the study or outcome level), and how this information is to be used in any data synthesis.	12
Summary measures	13	State the principal summary measures (e.g., risk ratio, difference in means).	12-13
Synthesis of results	14	Describe the methods of handling data and combining results of studies, if done, including measures of consistency (e.g., I^2) for each meta-analysis.	12-13

Section/Topic	#	Checklist Item	Reported on Page #
Risk of bias across studies	15	Specify any assessment of risk of bias that may affect the cumulative evidence (e.g., publication bias, selective reporting within studies).	12-13
Additional analyses	16	Describe methods of additional analyses (e.g., sensitivity or subgroup analyses, meta-regression), if done, indicating which were pre-specified.	12-13
**RESULTS**			
Study selection	17	Give numbers of studies screened, assessed for eligibility, and included in the review, with reasons for exclusions at each stage, ideally with a flow diagram.	2
Study characteristics	18	For each study, present characteristics for which data were extracted (e.g., study size, PICOS, follow-up period) and provide the citations.	3
Risk of bias within studies	19	Present data on risk of bias of each study and, if available, any outcome level assessment (see item 12).	
Results of individual studies	20	For all outcomes considered (benefits or harms), present, for each study: (a) simple summary data for each intervention group (b) effect estimates and confidence intervals, ideally with a forest plot.	
Synthesis of results	21	Present the main results of the review. If meta-analyses done, include for each, confidence intervals and measures of consistency.	
Risk of bias across studies	22	Present results of any assessment of risk of bias across studies (see Item 15).	
Additional analysis	23	Give results of additional analyses, if done (e.g., sensitivity or subgroup analyses, meta-regression [see Item 16]).	
**DISCUSSION**			
Summary of evidence	24	Summarize the main findings including the strength of evidence for each main outcome; consider their relevance to key groups (e.g., healthcare providers, users, and policy makers).	9-11
Limitations	25	Discuss limitations at study and outcome level (e.g., risk of bias), and at review-level (e.g., incomplete retrieval of identified research, reporting bias).	
Conclusions	26	Provide a general interpretation of the results in the context of other evidence, and implications for future research.	11
**FUNDING**			
Funding	27	Describe sources of funding for the systematic review and other support (e.g., supply of data); role of funders for the systematic review.	13

Supplementary 4. PRISMA Checklist
